# Nano-engineering of a thermoplastic compound with CuO nanoparticles for the development of safe antimicrobial and anti-biofouling fish cage nets

**DOI:** 10.1039/d5ra02611c

**Published:** 2025-06-20

**Authors:** Milad Mosallaei, Ella Kärkkäinen, Pekka Laurikainen, Chandrahaasan K. Soundararajan, Eetta Saarimäki, Mika Paajanen, Ilana Perelshtein, Kristina Ivanova, Eva Ramon, Tzanko Tzanov, Beatrice Negrini, Christian D'Abramo, Patrizia Bonfanti, Anita Colombo, Paride Mantecca

**Affiliations:** a VTT Technical Research Center of Finland Visiokatu4 33101 Tampere Finland milad.mosallaei@vtt.fi; b VTT Technical Research Center of Finland Kemistintie 3 Espoo FI-02150 Finland; c Bar-Ilan's Institute of Nanotechnology and Advanced Materials (BINA), Bar-Ilan University Ramat-Gan 5290002 Israel; d Department of Chemical Engineering, Universitat Politècnica de Catalunya Rambla Sant Nebridi 22 08222 Terrassa Spain; e University of Milano Bicocca, Department of Earth and Environmental Sciences, Research Centre POLARIS (Environmental Health & Sustainability) Milan Italy

## Abstract

The occurrence of biofouling is the most common problem that may arise on the surface of fish cage nets in freshwater and the marine environment. The issue not only induces structural damage to the netting because of the progressive increase in weight but also reduces the freshwater exchange and oxygen flow inside the cage, possibly leading to health problems for the farmed fish. Regular cleaning or inclusion of anti-fouling agents in the fish net cage material are typical methods to avoid biofouling growth and proliferation. Both are considered costly and pose health and environmental risks. In this study, a nano-engineered approach was adopted by embedding antimicrobial copper oxide (CuO) nanoparticles in the polyamide net fibers in order to prevent biofouling. The nano-engineered fish cage nets showed a strong anti-microbial effect, with up to a 6-log reduction of *S. aureus* and a 3-log reduction of *E. coli* in simulated seawater. Moreover, fine-tuning of the polyamide crystallinity level allowed for minor, within the range of 0.009 mg L^−1^ to 0.06 mg L^−1^, leaching of CuO nanoparticles into the aquatic environment. Aquatic toxicity, assessed in freshwater by monitoring mortality, malformations and hatching rates in zebrafish embryos exposed to different dilutions of samples leachates, over a 96 hour post-fertilization period, demonstrated a dose- and manufacturing-dependent embryotoxic effect, highlighting the importance of early-stage nanomaterial safety evaluation.

## Abstract 2

Monofilament produced *via* direct extrusion using a Berstorff twin-screw extruder with a PA6 matrix and CuO nanoparticles. Inset shows elemental mapping with relatively uniform nanoparticle distribution across the cross-section.

## Graphical abstract

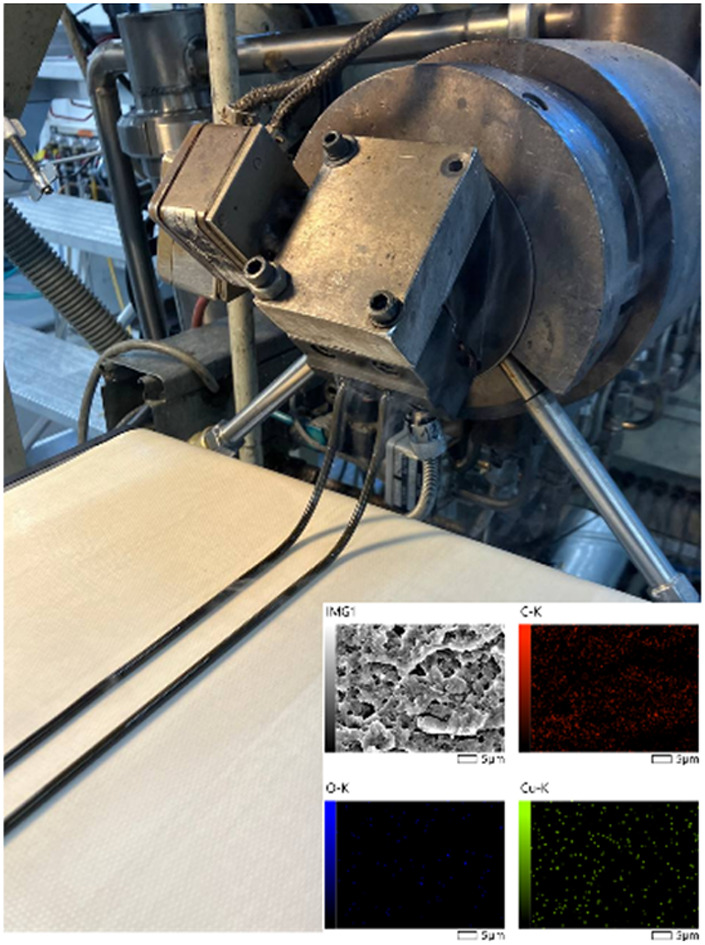

## Introduction

Aquaculture is a rapidly growing segment of industrial agriculture, and it is estimated that by 2030 over 52% of the seafood supply will derive from aquaculture.^1^ Aquaculture refers to the farming of fish, shellfish and other aquatic life by using different land- or water-based technologies enclosing the farm on a certain area.^2^ The disadvantages related to fish farming include the destruction of natural habitats, overharvesting, pollution, eutrophication and the distortion of the ecosystem caused by artificial feeding, and possible escape of non-native or genetically modified fish species.^3^ Furthermore, homogenous fish stock is more prone to infections than heterogenous wild-life fish cultures leading to increased need for antibiotics, disinfectants and pesticides.^3,4^

Fish cages not only contain farmed fish but also shield them from predators and the adverse effects of aquatic environments, *i.e.*, underwater fish farming cages are susceptible to strong currents and waves,^5,6^ and the accumulation of biofouling.^7,8^ Biofouling, the accumulation of microorganisms on the surface of the cage net, increases the mass and the hydrodynamic load on the net, causes clogging, and decreases the water and nutrient flow to the farmed fish. Biofouling is reduced by using different antifouling coatings and treatments.^9,10^ Aquaculture cages are made of various materials and structures; commonly, the cage consists of a frame and a mesh or netting. Polyamide (PA), polyethylene (PE), ultra-high molecular-weight polyethylene (UWPE), polyethylene terephthalate (PET), steel, copper and copper alloys are used as netting material.^11^ The cage frames can have a rigid, elastic/semi-flexible or a flexible structure, and be submersible or floating (non-submersible)^12,13^ depending on the farm site and farming conditions. Polyamide is a particularly popular choice for the fabrication of both inshore and offshore fish farm nets due to its weight, flexibility, high tensile strength, high abrasion resistance and overall durability.^14,15^ When it comes to the health of the fish, different antifouling approaches are employed including anti-fouler releasing coatings, non-sticking coatings, amphiphilic polymers, biomimetic materials, and different cleaning methods that create a harsh environment for biofoulers.^16^

The effect of copper as an antifouling agent has been extensively reviewed and studied, encompassing various technologies such as copper-based antifouling coatings,^7^ adhesive antifouling polyurethane films embedded with copper,^17^ copper-alloy nets,^18^ nano copper oxide in a hydrogel coating matrix,^19^ and commercially available shim tape.^17^

In the context of antimicrobial strategies, nanomaterials play a crucial role due to their distinct structural and chemical properties, leading to the emergence of a novel category of consumer goods known as nanoparticle-enabled products (NEPs).^20,21^ These nanoparticles (NPs) are used in a wide range of industries, including cosmetics, electronics, textiles, polymers, and pharmaceutics.^20,22,23^ Specifically, copper oxide nanoparticles (CuO NPs) have demonstrated remarkable antibacterial properties against both Gram-negative and Gram-positive pathogens due to high reactive oxygen species (ROS) generation and strong electrostatic interaction (positive surface charge) between bacteria and NPs.^24,25^ Despite their efficacy as antimicrobial and antibiofouling agents, concerns about NPs and NEPs biosafety persist. The same mechanism that make them a valuable alternative to conventional antibiotics also poses risks to non-target organisms exposed to unintentionally released or leached nanomaterials across various environmental compartments, including aquatic ecosystems.^26,27^ Therefore, it is essential to integrate safety assessments into the development of (nano)materials from the early design stage.

In this paper, we present a simple method to address durability, biofouling and antimicrobial challenges on fish cage nets by nano-engineering a polyamide 6 (PA6) thermoplastic polymer using CuO NPs. We investigated the antimicrobial properties, leaching behavior, and aquatic ecotoxicity of the net samples, both with and without NPs. In addition, the novelty of this study relies on the implementation of novel CuO NPs and the comparison of their functional and safety-related outcomes with those of commercial CuO NPs, whose toxicity has extensively been reported in literature.^28–31^ The reason was to investigate whether sonochemically-synthesized CuO NPs, which have been proved to be safe in terms of acute toxicity (*i.e.*, lethality^32^), could represent a safer alternative. Moreover, the modification of the extrusion process (*i.e.* extrusion speed) was considered as an additional parameter potentially affecting the hazardous behaviour of the new materials. To the best of our knowledge, no previous studies addressing these aspects for comparable materials are available in literature. Likewise, this approach is coherent with the new SSbD (Safe and Sustainable by Design) framework launched by the European Community to sustain the strategy for the production of safe and sustainable new chemicals and materials.^33–35^

## Materials and methods

### Materials

We used two grades of polyamide 6 (PA6) in this study, namely BASF Ultramid® B27E, a low-viscosity PA6 suitable for compounding and monofilament production, and PREXELENT™ PA601-10R (Premix PA6), a thermoplastic compound based on BASF PA6 with 10% tall oil rosin. *Staphylococcus aureus* (ATCC 25923) and *Escherichia coli* (ATCC 25922) were obtained from the American Type Culture Collection (ATCC® LGC Standards, Spain). Baird-Parker agar, Coliform ChromoSelect agar, Mueller Hinton broth (MHB), phosphate-buffered saline (PBS) was purchased from Sigma-Aldrich (Spain). Analytical-grade reagents, embryo solution (ES) salts and 3-amino-benzoic acid ethyl ester (MS222) were purchased by Merck KGaA (Darmstadt, Germany). Embryo solution composition is 100 mg L^−1^ NaHCO_3_, 100 mg L^−1^ Instant Ocean salt, 190 mg L^−1^ CaSO_4_. Instant Ocean salt was purchased from Aquarium Systems (Sarrebourg, France).

### CuO nanoparticles preparation

Sonochemically-synthesised CuO NPs were developed in this study (D CuO NPs). D CuO NPs were prepared by dissolving 0.6 g of copper acetate in the 300 mL aqueous solution, yielding a copper precursor concentration of approximately 0.02 M. The solution was then transferred to a sonication flask, placed in a temperature-regulated bath at room temperature. Sonication was performed using a Ti horn probe sonicator (Ultrasonic Disruptor, 20 kHz, 675 W, 2.5 cm probe diameter) operated at 35% amplitude. When the temperature reached 60 °C, an aqueous solution of ammonia was gradually added to adjust the pH to 8. The sonication was conducted for 30 minutes. After the reaction, the resultant solution was centrifuged, washed with water and ethanol and dried at 60 °C overnight. A commercially available CuO nanopowder with an average particle size below 50 nm was also obtained from Sigma-Aldrich as a control NPs for comparison with the one developed in this study.

### Specimen preparation

Two different series of samples were fabricated for this study: monofilaments and mini-injection moulded pieces. Monofilaments were used to fabricate fish cage nets. However, mini-injection molded pieces and cast films were employed for the initial antimicrobial and leaching analysis.

CuO NPs were incorporated during compounding of both monofilaments and mini-injection molded pieces, while NPs were not added to the cast film composition. Cast films were coated with CuO NPs separately. Table 1 shows the formulation recipe for preparation of the mini-injection pieces, where the recipes 009 and 015 were later used for the fabrication of monofilaments.

**Table 1:** Compounding recipes for injection moulded samples (001–016) and monofilaments (009, 015)

Sample code	Materials concentration (%)			
	BASF PA6	Premix PA6	D CuO NPs	Commercial CuO NPs
001	80	20	0	0
002	60	40	0	0
003	40	60	0	0
004	20	80	0	0
005	98	0	2	0
006	78	20	2	0
007	58	40	2	0
008	38	60	2	0
009	18	80	2	0
010	0	98	2	0
011	98	0	0	2
012	78	20	0	2
013	58	40	0	2
014	38	60	0	2
015	18	80	0	2
016	0	98	0	2

The materials were accurately weighed, pre-mixed using a SpeedMixer® DAC 150 (Hauschild GmbH, Hamm, Germany) at 2500 rpm for 30 seconds, and dried in an oven at 100 °C for two hours. The moisture level was analyzed with a water-selective moisture analyzer (Brabender Aquatrack-V, Duisburg, Germany) to ensure low moisture content in the compound before further processing (<800 ppm). The materials were then sealed in aluminum bags filled with argon to prevent moisture uptake before the subsequent processing steps. For the next step, melt-blending was conducted using a DSM Xplore mini-scale twin-screw extruder (Xplore Instruments BV, Sittard, The Netherlands) at a temperature of 230 °C, screw speed of 100 rpm, and mixing time of 4 minutes. After melt-blending in the micro-compounder, the compounds were transferred in the molten state to a Thermo-Haake minijet injection molding machine (Thermo Fisher ScientificTM, Massachusetts, USA) to prepare small pieces, with approximate dimensions of 25 × 25 × 2 mm^3^ (injection temperature 230 °C, mold temperature 40 °C, injection time 10 seconds, pressure 1000 bar, hold time 5 seconds).

**Fig. 1:**
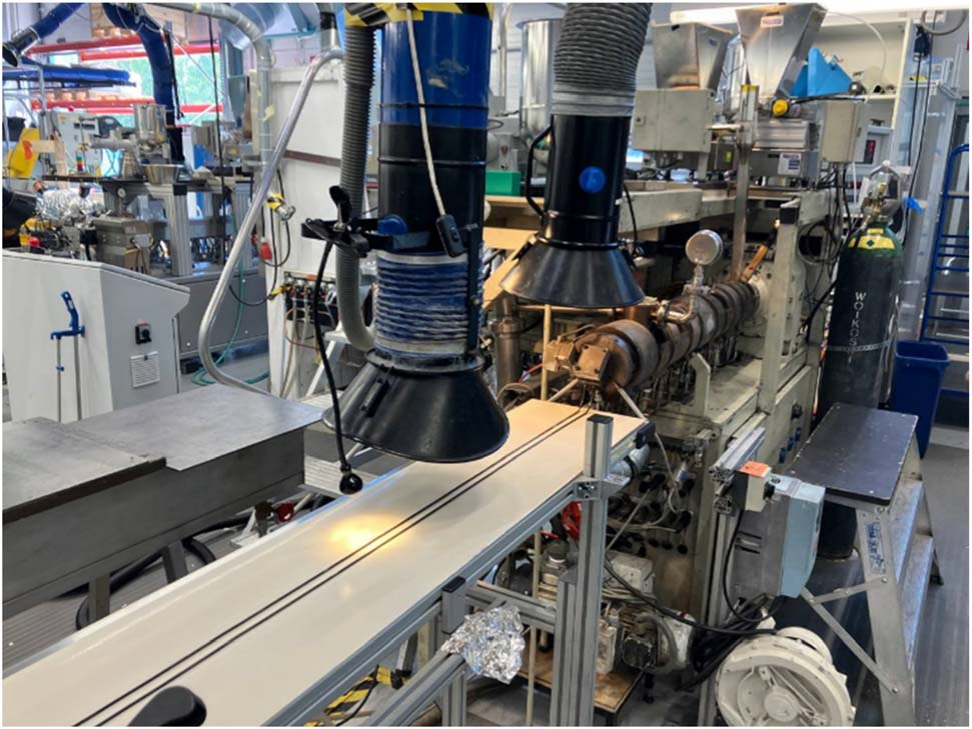
Production line of monofilaments by Berstorff twin-screw extruder.

The monofilament production began with the preparation of a masterbatch comprising 10 wt% CuO mixed into the BASF Ultramid polyamide. The masterbatch was obtained using a Brabender Plasti-Corder single-screw extruder with a mixing element in the homogenization zone of the screw. The purpose of the masterbatch was to improve workplace safety for compounding with CuO NPs embedded in polymer matrix facilitating risk-free gravimetric feeding. The 10 wt% mixing ratio was selected as based on the preliminary mixing trials, this amount is easily dispersed in the melt and facilitates simple compounding as the masterbatch can be mixed directly with the Premix PA6 material to create the final compound with 2 wt% CuO. The monofilaments were prepared by direct extrusion from the compounding process in a Berstorff twin-screw extruder as shown in Fig. 1. Data from preliminary trials with Ultramid PA were used to select two different processing parameter configurations – denoted here simply as fast (10 kg h^−1^) and slow (3 kg h^−1^) – in order to obtain two different degrees of crystallinity for the polymer material. For the preliminary trials with Ultramid PA crystallinities of 28% and 33% were detected by differential scanning calorimetry DSC (TA Instruments DSC 2920) for the fast and slow process configurations, respectively.

### Monofilament characterization by scanning electron microscope (SEM) and production demonstration

For imaging and compositional analysis of CuO particles at the cross-section of the monofilament, the samples were immersed in liquid nitrogen for 2–3 minutes and subsequently fractured mechanically. They were then sputter-coated with a 5 nm layer of Au–Pd and analysed using a TESCAN AMBER X system. An acceleration voltage of 5 keV and low beam current were employed to resolve the fine nanoparticles in both imaging and compositional analysis. To ensure the distribution quality of the CuO nanoparticles within the PA6 matrix, EDS elemental mapping was conducted using a JEOL JSM-IT710HR FE-SEM on cross-sections of monofilaments prepared with either commercial or D CuO. For this purpose, the monofilaments were cryo-fractured in liquid nitrogen and coated with a 5 nm layer of gold to enhance conductivity. EDS elemental mapping was performed using backscattered electrons at an accelerating voltage of 15 kV and a working distance of 10 mm. SEM characterization followed the methodological guidance of previously reported literature.^36–39^

### Demo preparation

**Fig. 2:**
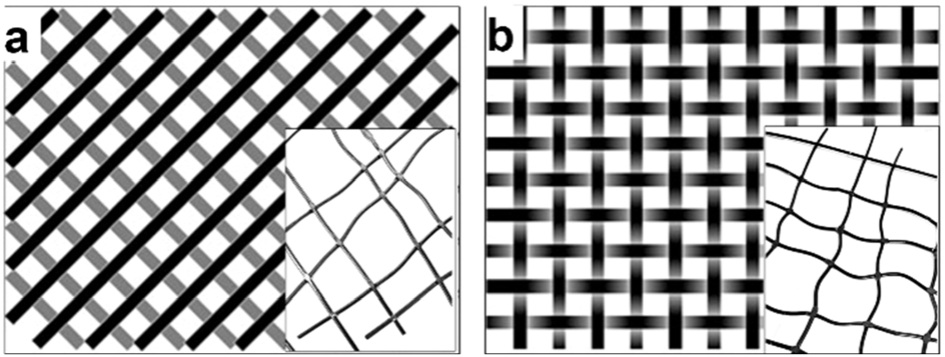
Schematic presentation of the (a) diagonal structure with half of the monofilaments in one layer, and half in the other one, and the (b) woven structure with intertwined monofilaments. The heat pressed monofilament nets: the (inset a) diagonal structure.

The filaments prepared with the Berstorff twin-extruder were cut and arranged into two different forms for a production demonstration: a diagonal net of two layers (Fig. 2a) and a woven structure (Fig. 2b) of size 40 × 50 cm with a mesh size of 5 × 5 cm, approximately. A tabletop heat press Color-King, CKB5-2 was used to form the net links by melting the joints. The diagonally built net was pressed at 220 °C for 60 seconds, forming some weak links. The woven structure was pressed with the same temperature and pressure, but for an additional 60 seconds. The woven structure added stability and allowed for easier handling.

Both-sides heating of the monofilaments led to their partial melting. The monofilaments softened in both the diagonal structure (inset of Fig. 2a) and the woven structure (inset of Fig. 2b), leaving the monofilament pattern bent and with uneven mesh sizes. Melting-together happened at the joints, where the nets were thickest. The woven structure provided more dimensional stability than the diagonal structure. The additional 60 seconds of pressing complemented the integrity of the woven filaments making it a more reliable structure.

**Fig. 3:**
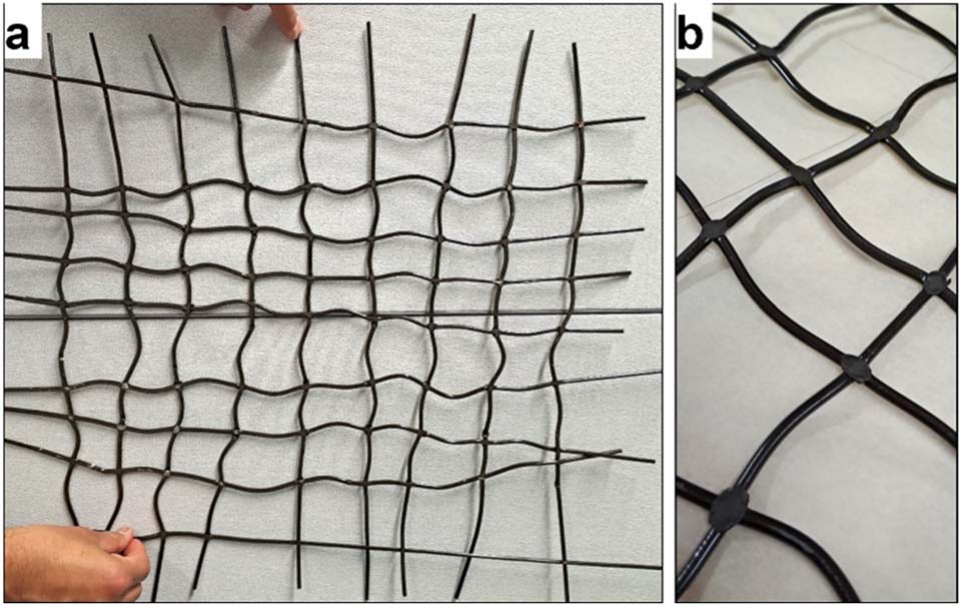
Final demonstrators, the (a) woven structure with some dimensional distortion. The joints seemed to have melted well (b).

The final net demonstrator is presented in Fig. 3a. Fig. 3b shows the joints up-close, demonstrating how the melting of the monofilaments happened at the thickest points without melting the round cross section of the filaments.

### Antimicrobial activity

The antimicrobial activity of the mini-injection moulded samples was evaluated using the contact killing method. Single colonies of *E. coli* and *S. aureus* were grown in 5 mL of sterile MHB at 37 °C and 230 rpm overnight. Cultures were then diluted with PBS, pH 7.4 to an absorbance of 0.01 at 600 nm. Subsequently, 0.1 mL of bacterial suspension were placed on the top of each 1 × 1 cm sample in a 24-well sterile plate and the samples were incubated for 24 h at 37 °C. Following incubation, 1 mL sterile PBS was added to the samples and the plate was placed in an ultrasonic bath for 10 min to recover the bacterial cells attached to the surface. The live bacteria were quantified by plating serial dilutions of the bacterial suspension on Coliform and Baird-Parker agar plates for *E. coli* and *S. aureus*, respectively, followed by colony-forming units (CFU) enumeration.

The antibacterial activity of monofilaments was assessed following the same protocol with some modifications due to the three-dimensional geometry of the samples. Briefly, the monofilaments samples (3 × 1 cm) were incubate in 1 mL sterile PBS containing *S. aureus* and *E. coli* bacteria at absorbance of 0.01 and incubated for 24 h at 37 °C under gentle shaking. Afterwards, the surviving bacteria were quantified by plating on agar plates as already described.

The durability of the developed materials was studied measuring the antibacterial activity of the samples after three weeks incubation in simulated marine water (SMW). Samples (1 × 1 cm) were incubated in 10 mL SMW containing ammonium sulfate, potassium dihydrogen phosphate, sodium bicarbonate and trace metals (Fe, Cu, Co, Zn, Mn, Ni and Mg) at concentration of 32 ppm with agitation (180 rpm) and room temperature (RT). After incubation, the samples were washed with sterile distilled water, air-dried under sterile conditions and subjected to antibacterial evaluation as described before.

### Leaching test for characterization and ecotoxicity tests

The leachates from pristine (reference) mini-injected pieces and samples containing the two different types of NPs (commercial or D CuO NPs) were obtained by stirring the material (1 × 1 cm) in 10 mL ES. To obtain leachates from monofilaments made of reference, D CuO NPs and commercial CuO NPs, each produced with slow and fast processing parameters, samples were immersed in embryo solution with a length/volume ratio of 1 cm/1 mL. Stirring lasted three weeks at a speed of 100 rpm at RT.

Six milliliters of each leachate were collected for Inductively Coupled Plasma Optical Emission Spectrometry (ICP-OES) analyses to quantify the release of copper ions and the total copper content. The samples were ultracentrifuged with centrifuge tubes VIVASPIN20 (Sartorius Stedim Biotech GmbH, Goettingen, Germany) with molecular weight cut-off 10 kDa (4000 × *g*, 25 °C, 10 minutes). Ultrafiltrates were then analyzed (PerkinElmer Optima 7000DV, PerkinElmer, Santa Clara, CA). The remaining leachates were used as exposure medium for zebrafish embryos in the Fish Embryo acute Toxicity (FET) test.^40^

### Fish husbandry breeding

Adult zebrafish (AB wild-type strain) were sourced from the European Zebrafish Resource Center (Karlsruhe Institute of Technology, Germany) and bred at the University of Milan-Bicocca facility (ethical approval ATS MetroMilano Prot. n. 0020984–12/02/2018). The breeding took place in a ZebTec Active Blue circulating system (Tecniplast, Buguggiate, Italy).

The breeding conditions were 28 °C, pH 7.5, conductivity of 500 μS and 14:10 h light–dark cycle. The breeding pairs were fed two times a day with a Zebrafeed diet (Sparos Lda, Olhão, Portugal). The evening before mating and spawning, breeding tanks were used to maintain separate males and females and in the morning the barrier was removed to allow the mating. Eggs were immediately collected, and the fertilized ones were selected with a stereomicroscope (Zeiss, Germany).

### Fish embryo acute toxicity (FET) test

The acute toxicity of leachates from the mini-injected pieces and from the monofilaments was assessed for zebrafish embryos with the FET test.^40^ In particular, the FET test here performed was exploited as a valuable screening tool for identifying the potential hazards of the materials, taking advantage of its ability to quickly flag materials with acute toxic effects at early developmental stages, and to provide essential preliminary data, thus enabling prioritization for further testing during the material design. Accordingly, the aim was to perform a hazard screening and early-phase material safety evaluations.

The solutions tested for the mini-injected pieces were the “stock” leachates (*i.e.*, leachates directly obtained after the three weeks leaching conditions) and five dilutions of the 3 weeks stock leachates: 1 : 5, 1 : 10, 1 : 25, 1 : 50, 1 : 100. Monofilaments leachates were tested both as stock dilution and four decreasing concentrations from stock, *i.e.* 1 : 2.5, 1 : 5, 1 : 10, 1 : 25.

Freshly fertilized eggs (<3 hours post-fertilization, hpf) were randomly selected after spawning and placed individually in 24-well plates, in which 20 embryos were exposed to 1.5 mL of leachate dilution either from the pristine mini-injected pieces or monofilaments and mini-injected pieces or monofilaments moulded with commercial or D CuO NPs (treatment groups). In each plate four embryos were used as the negative control group (ES medium). Multiwells were placed in a thermostatic chamber at 26 ± 0.5 °C, under static conditions. The test was run in independent triplicates for all the conditions.

All exposure groups were screened with a stereomicroscope for lethal and sub-lethal endpoints at 24, 48, 72 and 96 hpf.^40,41^ Lethal endpoints such as coagulation of fertilized eggs, lack of somite formation, lack of detachment of the tail-bud from the yolk sac and lack of heartbeat were checked every day starting from 24 hpf to evaluate acute toxicity. Hatching and malformations rates were additionally recorded as sublethal endpoints for embryos exposed to monofilaments' leachates starting from 48 hpf and at 96 hpf, respectively.

### Statistical analysis

Statistical analysis for the antibacterial tests were performed using the GraphPad Prism (version 10). One-way analysis of variance (ANOVA) followed a *post hoc* Tukey's test was applied. All reported data were presented as the mean value ± standard deviation (SD).

*In vivo* tests data were analysed by a one-way analysis of variance (ANOVA). All statistical analyses were conducted with a confidence level of at least 95% using IBM SPSS Statistics 26 Software. The data were presented as the mean ± standard error (SE). Statistical significance was defined as *p*-values less than 0.05 and 0.01.

## Results and discussion

### CuO NPs characterization

The morphology and size of the D CuO NPs were examined by high resolution transmission electron microscopy (HRTEM) JEOL 2100 (Peabody, MA, USA). The crystallographic phase of the D CuO NPs was confirmed by using X-ray diffraction (XRD) Bruker Inc. (Germany) AXS D8 ADVANCE (voltage 40 kV, monochromatic Cu Kα radiation (*λ* = 0.15418 nm)) and selected area diffraction pattern.

**Fig. 4:**
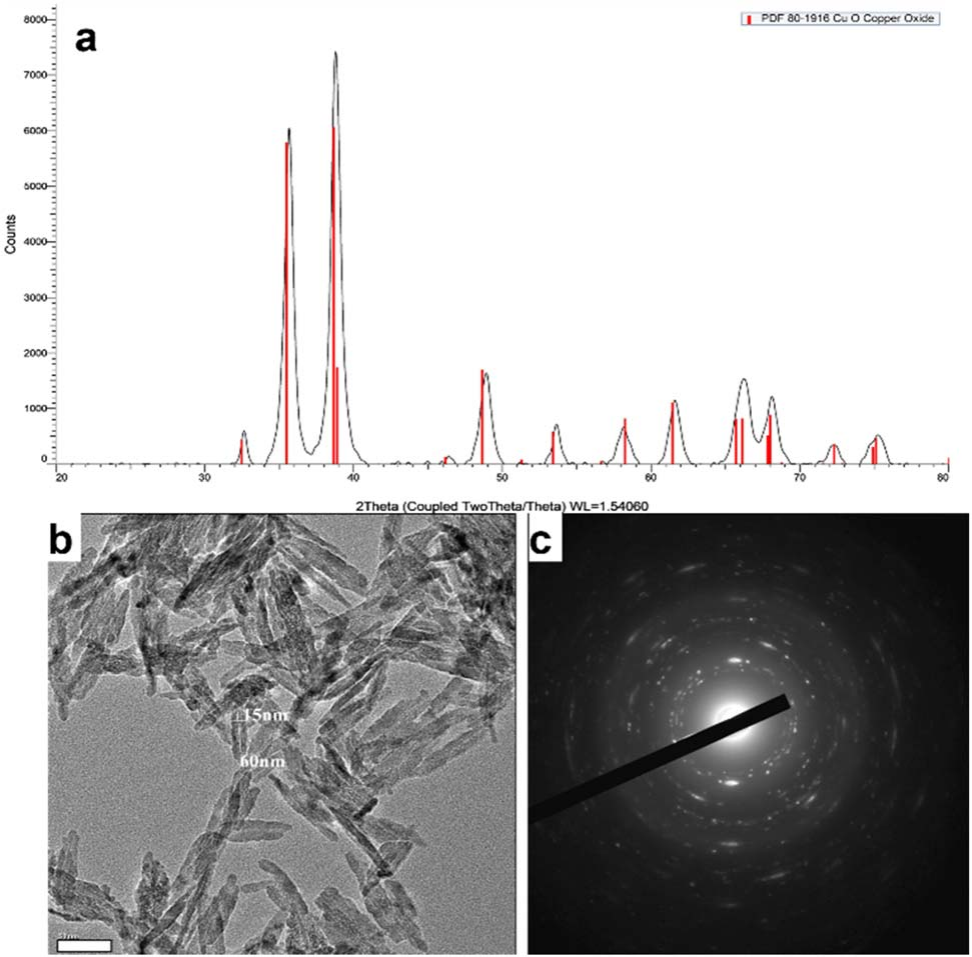
D CuO NPs characterization. (a) XRD pattern, (b) HRTEM image, and (c) SAED of D CuO NPs. Scale bar in (b): 50 nm.

The crystallographic phase of the prepared D CuO NPs was analyzed by XRD. As denoted in Fig. 4a, the XRD pattern clearly demonstrated consistency with the JCPDS data (80-1916) of the CuO with a monoclinic phase.^42^ The morphological studies and particles size of the synthesized NPs were performed by HRTEM analysis, which showed in Fig. 4b the NPs size between 50–150 nm.^43^ Besides, the crystalline nature of the particles was confirmed by the selected area diffraction (SAED) pattern (Fig. 4c).^44^

### Nano-enabled products physico-chemical characterization

SEM analyses were performed on monofilaments to verify the NPs distribution on the final product as the monofilament are used for the production of the final product.

**Fig. 5:**
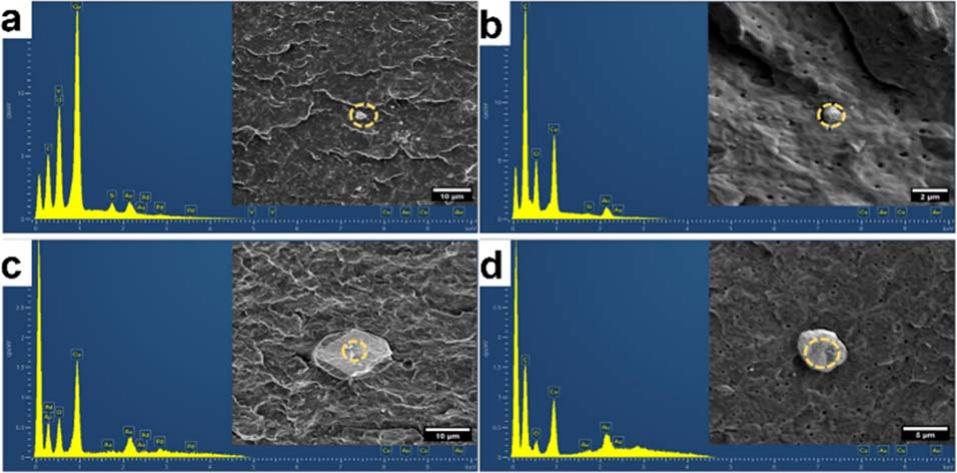
Cross-sectional and EDS spectrum of monofilament prepared by D CuO NPs; (a) slow, (b) fast and commercial CuO (c) slow and (d) fast processing configuration. Scale bars: (a and c) 10 μm, (b) 2 μm, (d) 5 μm.

The obtained EDS spectra are mostly from particles of relatively large size (Fig. 5). This is because of the large drift associated with imaging smaller particles. The drift makes it difficult to obtain X-ray signal for reasonable amount of time.

**Fig. 6:**
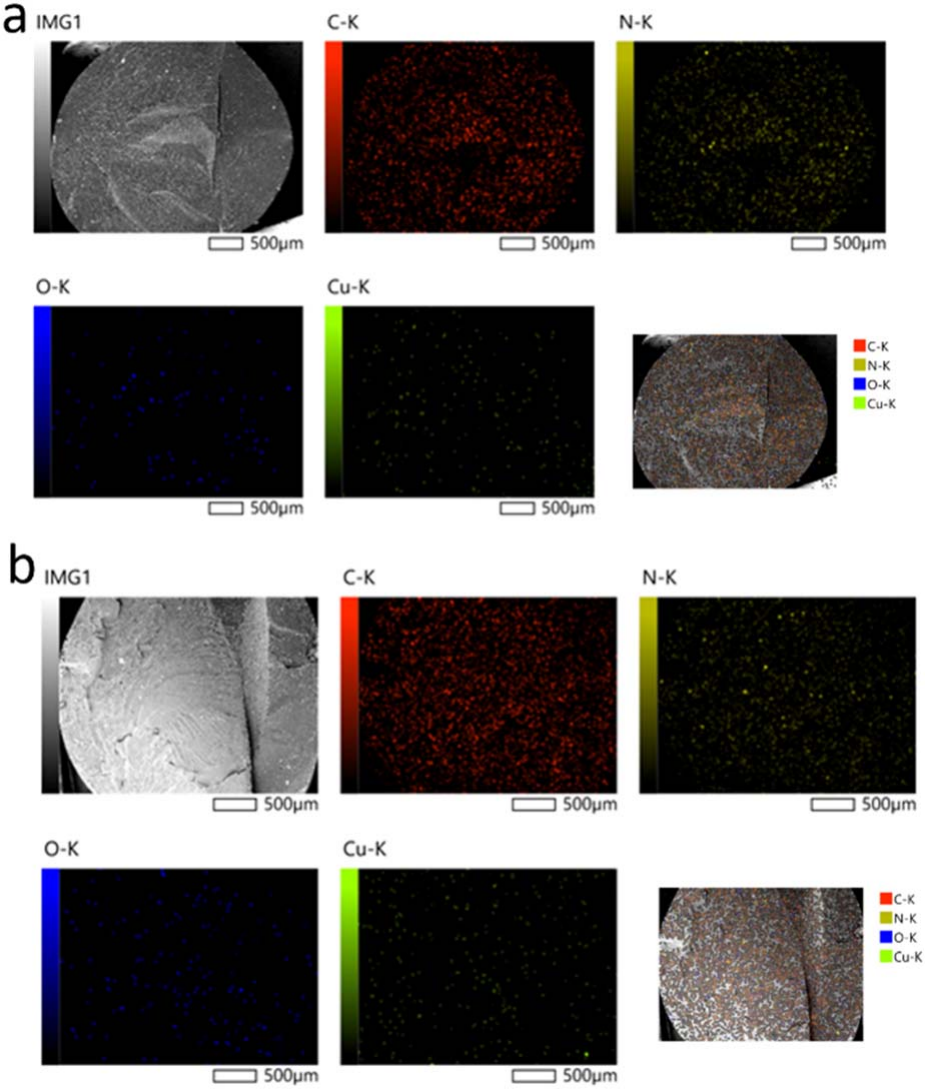
EDS elemental mapping of CuO NPs dispersed in the cross-section of polyamide 6 monofilaments prepared at a throughput of 3 kg h^−1^ using: (a) commercial, (b) D CuO NPs.

Fig. 6(a and b) shows the distribution maps for copper (Cu), oxygen (O), nitrogen (N), and carbon (C), confirming a relatively uniform dispersion of the nanoparticles. Although the size of individual nanoparticles ranges from 50 to 150 nm, they appear larger (a few micrometers) in the images, most likely due to local clustering. However, the distribution of these clusters remains relatively uniform.

Copper ions (Cu^2+^) release from mini-injected pieces and monofilaments polymeric matrix was analyzed. The amount of total copper (given by both Cu^2+^ and CuO NPs) was also evaluated for monofilaments. The results of mini-injected pieces ICP-OES characterization are reported in Table 2.

**Table 2:** Cu^2+^ concentration (mg L^−1^) in leachates from reference and CuO (D or commer-cial) mini-injected pieces after a leaching test of three weeks

Sample	Cu^2+^ concentration, mean ± SD (mg L^−1^)
Reference	−0.003 ± 0.007
D CuO	0.006 ± 0.011
CuO commercial	0.021 ± 0.004

As expected, in the reference pieces-derived leachates no Cu^2+^ release was found. Compared to this result, a relatively low release of copper in the ES medium was observed after a 3 week leaching test. Particularly, an increased release from pieces moulded with commercial CuO NPs was highlighted when compared to the D NPs-enabled ones. In the commercial's leachate, Cu^2+^ was 3.5 times higher than the Cu^2+^ from D NPs-enabled mini-injected pieces. The results of monofilaments ICP-OES characterization are reported in Table 3.

**Table 3:** Cu^2+^ concentration (mg L^−1^) in leachates from reference and CuO (D or commercial) monofilaments after a leaching test of three weeks. The mean results were cal-culated subtracting the mean values of the respective reference materials

Sample	Cu^2+^ concentration, mean ± SD (mg L^−1^)	Total Cu concentration, mean ± SD (mg L^−1^)
CuO commercial 3 kg h^−1^	0.009 ± 0.002	0.021 ± 0.000
D CuO 3 kg h^−1^	0.019 ± 0.004	0.034 ± 0.001
CuO commercial 10 kg h^−1^	0.057 ± 0.005	0.225 ± 0.004
D CuO 10 kg h^−1^	0.029 ± 0.007	0.105 ± 0.013

A minimum amount of copper was detected in the reference materials of both process configurations, possibly due to contamination during the manufacturing process, *e.g.* copper-based anti-seize grease used in the extruder assembly. Because of this, the reported values are the result of a subtraction of the average Cu^2+^ found in references to the leachates derived from NPs-enabled products. All Cu^2+^ amounts are in the range of μg L^−1^ (from 9 to 60, *ca.*). A higher release of ions was detected in the fast process at 10 kg h^−1^ throughput, particularly in the leachates from monofilaments enabled with commercial CuO NPs. As regards the monofilaments from the slow process at 3 kg h^−1^, Cu^2+^ is found with double values in the leachates enabled with D NPs. Comparing the two CuO NP types with the fast process, only approximately 50% of Cu^2+^ content was detected from the D NPs, compared to the commercial NPs-enabled sample. Total dissolved copper ranges from 20 to 230 μg L^−1^, with monofilaments extruded at 10 kg h^−1^ releasing an order of magnitude more copper than those extracted at a lower rate. The same trend observed for Cu^2+^ is maintained overall.

In general, the polymeric matrix extruded at a 3 kg h^−1^ throughput appears to better retain commercial CuO NPs, while changes in crystallinity appear to affect the two CuO NPs leaching rates differently. In the filaments prepared with the fast process configuration the D NPs are retained more effectively. Nevertheless, both types of NPs are less retained by the 10 kg h^−1^ extruded monofilaments. Indeed, lower pulling speeds during extrusion are known to produce a more crystalline and tighter polymer structure, while higher pulling speeds yield a more amorphous and open composition.^45^ Thus, polymers doped with metal oxide nanomaterials can be more or less prone to mobilize and release NPs and ions. Moreover, the polymers doped with commercial CuO NPs released more Cu (both in soluble and particulate form) than the one doped with D CuO, confirming that also the P-chem characteristics of the NPs are able to influence the leaching from the matrix. This may be attributable to the different shape of the nanoforms, being the commercial CuO NPs round-shaped, while the sonochemical ones leaf-shaped.^46^

#### Antimicrobial activity

The antibacterial activity of the mini-injection moulded samples, made of BASF or Premix polymers with varying concentration of D CuO NPs (0–3% (w/v)), was evaluated against the Gram-positive *S. aureus* and Gram-negative *E. coli* using a contact killing assay. This method was selected since the CuO NPs are embedded in the polymer matrix preventing them from diffusion to the environment to kill bacteria. As a result, the antimicrobial activity is most probably confined to the surface of the material rather than being attributed to NPs release.

**Fig. 7:**
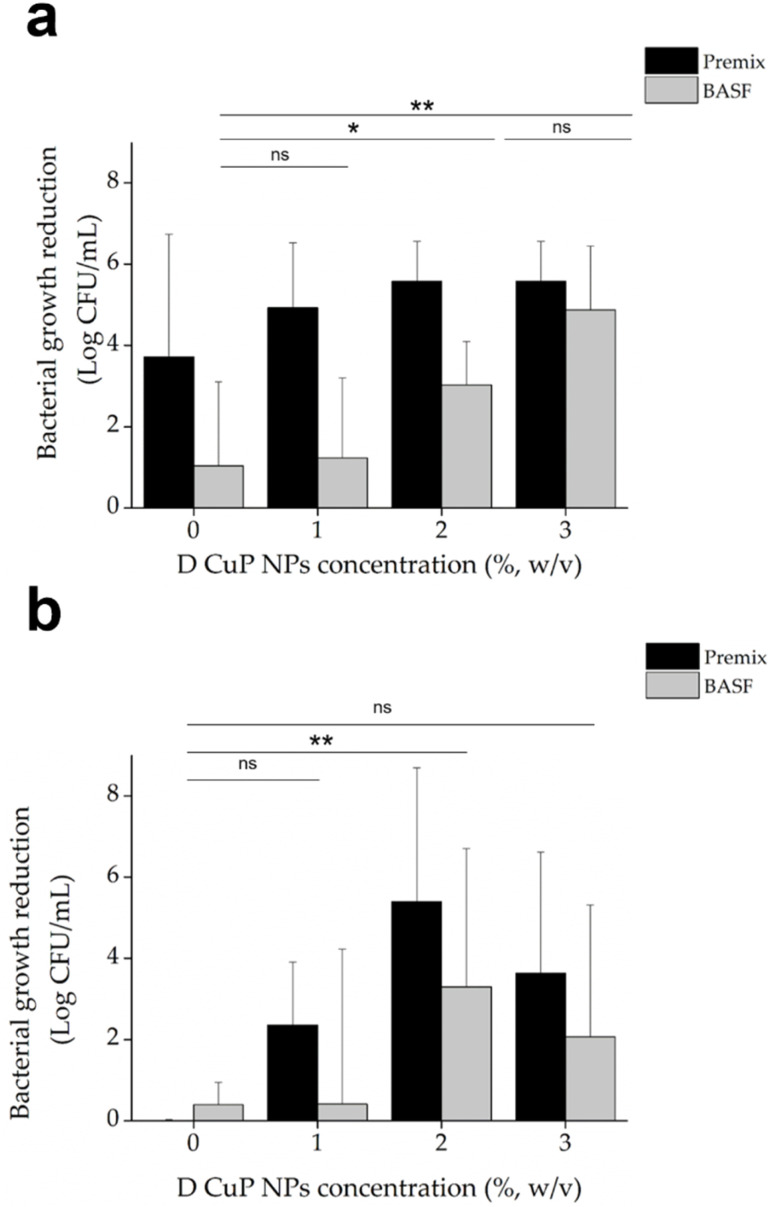
Antibacterial activity of mini-injection molded samples made of Premix (black bars) or BASF (grey bars) polymers without and with D CuO NPs at varying concentrations (1, 2, and 3%, w/v) against *S. aureus* (a) and *E. coli* (b). *p < 0.05, **p < 0.01 *vs.* control (one-way ANOVA, post Tukey's Multiple Comparison test, *n* = 4).

The antibacterial tests demonstrated that samples loaded with 2% CuO NPs yielded strong bactericidal effect against *S. aureus*, reducing the live bacterial load by more than 5 logs, regardless of the polymer type. Increasing the CuO NPs concentration beyond 2% did not result in any further significant improvement in the antibacterial activity. Premix PA6 samples without CuO NPs also exhibited notable inhibitory effects on *S. aureus*, an effect that was not observed for BASF polymer (Fig. 7a). This inherent antibacterial activity of Premix polymer itself is due to additives included in the polymer formulation.^47–49^

In the case of *E. coli*, the pristine Premix sample did not show bactericidal activity, but the addition of CuO NPs (at 2% (w/v)) led to significant improvement in the bactericidal activity. Approximately 3 logs reduction was obtained for the BASF samples and 5 logs for Premix samples, compared to the 1% CuO NP samples, which showed live count reductions of 0.4 logs and 2 logs, respectively. However, increasing the NP concentration to 3 wt% did not result in antibacterial enhancement, as observed also for *S. aureus*. This suggests that the 2% CuO NPs concentration is optimal for maximum antibacterial effectiveness against both *E. coli* and *S. aureus* (Fig. 7b). Consequently, the 2% CuO NPs concentration was set as a constant for the subsequent experiments, and new samples were prepared with varying ratios of the two PA6 polymers.

**Fig. 8:**
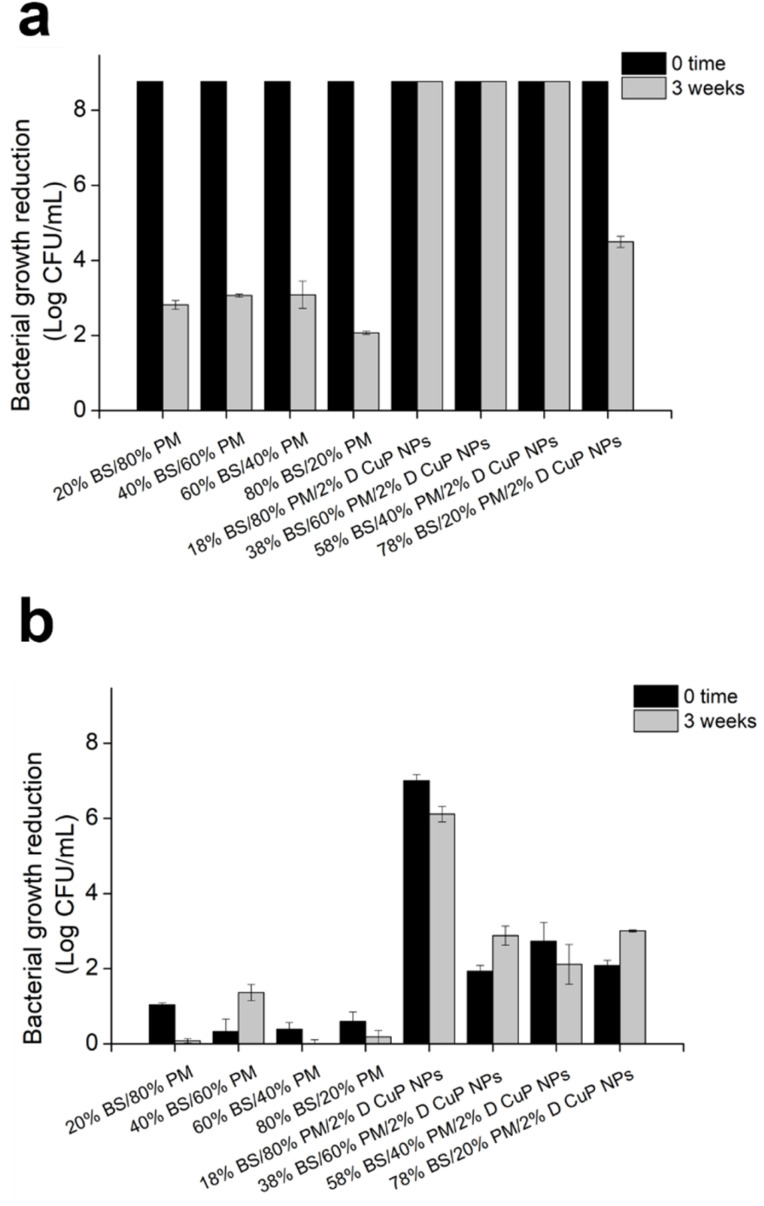
Antibacterial activity of mini-injected molded samples prepared at varying BASF (BS) and Premix (PM) polymers ratios and 2% D CuO NPs towards *S. aureus* (a) and *E. coli* (b) at time 0 (black bars) and after 3 weeks three-weeks incubation in SMW.

Among all the samples developed, those composed of 18% BASF PA6, 80% Premix and 2% CuO NPs demonstrated strong antibacterial activity against both bacterial strains at time zero (Fig. 8). Importantly, this effect was sustained even after three weeks incubation in simulated marine environment. In contrast, the control samples without CuO NPs, showed similar effects on *S. aureus* at time zero but experienced a significant reduction in antibacterial activity. This initial effect is probably attributed to the tall oil rosin present as an additive in Premix PA6, which constitutes 80% of the blended samples. Tall oil rosin has been employed to enhance the antibacterial performance of various materials, showing stronger effect against Gram-positive bacteria, such as *S. aureus*, while exhibiting lower efficacy against Gram-negative bacteria.^50^ However, this effect diminished over time due to the potential release or washout of the oil from the material in simulated marine environment. The incorporation of antibacterial CuO NPs significantly enhanced the antibacterial performance of the materials, as demonstrated by the sustained activity against both Gram-positive and Gram-negative bacteria over time.

**Fig. 9:**
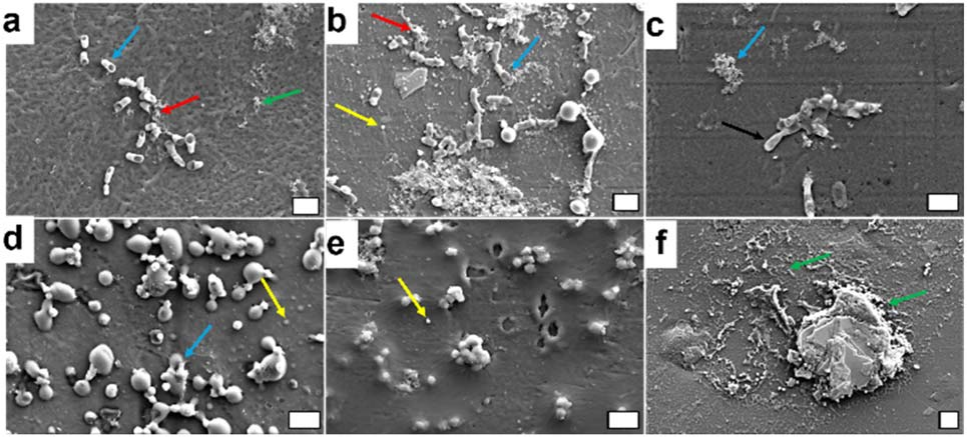
Scanning electron microscopy (SEM) images of *E. coli* and *S. aureus* cells exposed to different CuO-based materials. *E. coli* cells on D CuO NPs containing materials (a), *E. coli* cells on commercial CuO NPs containing materials (b), *E. coli* on pristine materials (c), *S. aureus* cells on D CuP NPs containing materials (d), *S. aureus* cells on commercial NPs containing materials (e), and *S. aureus* cells on pristine material (f). (Color-coded arrows indicate blue – cellular damage, red – cellular debris, black – intact bacterial cells, yellow – NPs), green – artifacts. Scale bars: 2 μm.

SEM images of the samples containing 18% BASF PA6, 80% Premix and 2% D CuO NPs or commercial CuO NPs, prior durability tests, further validated their antibacterial performance against *E. coli* and *S. aureus* bacteria (Fig. 9). These findings were consistent with the results obtained using bacteria plate counting method. Adherent *E. coli* cells with morphological perturbations consistent with the killing mechanism of CuO NPs were observed. In the samples containing D CuO NPs, the cells exhibited structural damage including visible holes, likely attributed to alteration of bacterial membrane caused by the NPs, along with cellular debris resulting from membrane leakage and subsequent cell death (Fig. 9a). A comparable trend was observed with the samples prepared with commercial CuO NPs (Fig. 9b). The SEM images showed bacterial cells with altered morphology and NPs distributed unevenly across the surface. This uneven distribution could be due to inhomogeneous blending of the polymers with the NPs, or to the more superficial localization of the NPs. The latter may contribute to reduced stability, as smaller NPs, such as the commercial CuO NPs, are more prone to migration or release in liquid medium. However, such behavior was not directly observed in the SEM images. In pristine samples, intact *E. coli* cells forming aggregated were present (Fig. 9c). Additionally, some artifacts and cellular morphological changes were apparent, even at time zero (prior to incubation in the simulated marine environment). These changes suggest a pre-existing antibacterial effect, likely due to the additive present in the pristine polymer matrix, which aligns with the results obtained through the plate count method.

For the samples incubated with *S. aureus*, different observations were made based on the type of CuO NPs and the pristine polymer samples. In the case of D CuO NPs, we clearly observed cells with altered morphology, probably caused by the direct contact with the NPs on the surface. Additionally, some NPs were visible on the surface (Fig. 9d). This supports the hypothesis that the distribution of NPs in the blends is uneven, resulting in areas with higher concentrations of embedded and others with surface-localized NPs. For the commercial CuO NPs, it is challenging to conclude whether the observed structures are cellular debris or NPs. This ambiguity arises because most of the structures in the image are spherical, small, and likely coated with polymer layer, whereas some regions present aggregates of NPs rather than deformed cells. The sample preparation procedure prior to SEM imaging could also influence this observation. The results for the pristine sample follow a similar tendency. The SEM images primarily revealed artifacts from the material and likely some cellular debris but did not show intact cocci with a spherical shape measuring 1–2 μm. This finding aligns with the fact that at time zero, the pristine samples exhibited a strong bactericidal effect on this Gram-positive bacterium (Fig. 7).

**Fig. 10:**
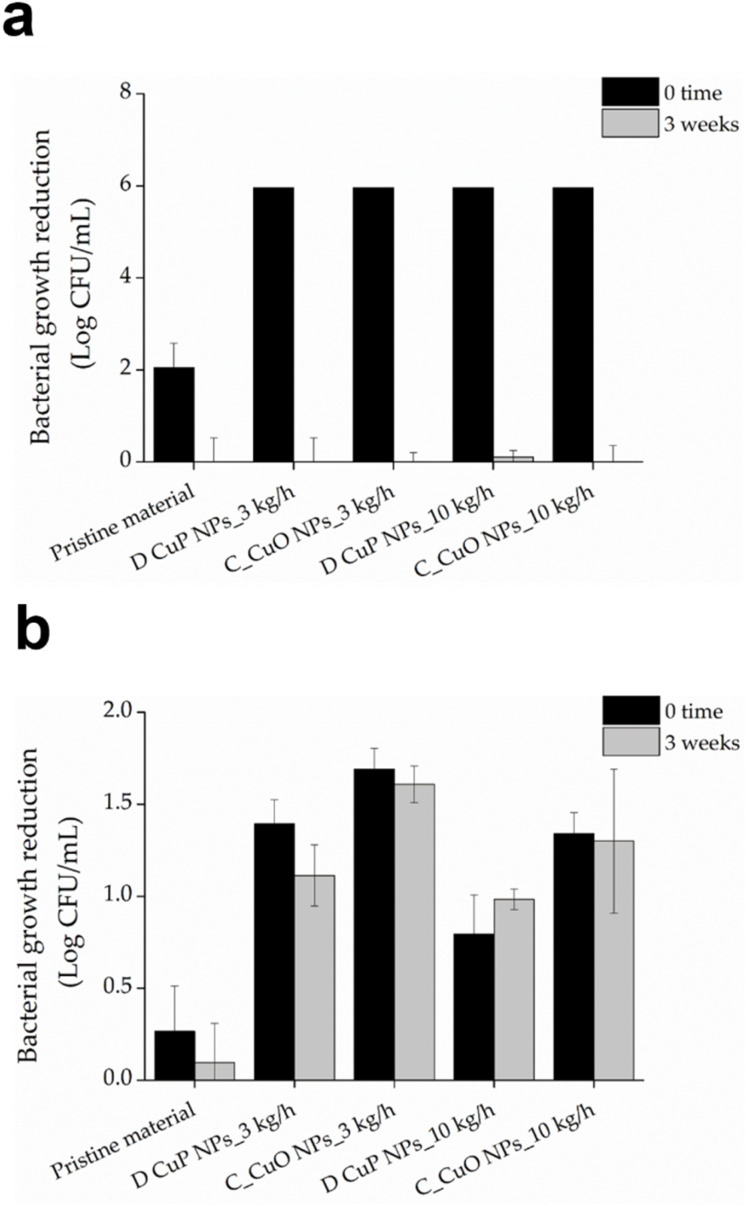
Antibacterial activity of monofilaments for fish cage net production, composed of 18% BASF PA6, 80% Premix, and 2% D CuO NPs or commercial CuO NPs and produced at 3 or 10 kg h^−1^ extrusion speed, against of the *S. aureus* (a) and *E. coli* (b) at time zero (black bars) and after three-weeks (grey bars) incubation in SMW.

Additionally, monofilaments for fish cage net production, composed of the same matrix formulation (18% BASF PA6, 80% Premix, and 2% CuO NPs), were produced using two different sets of processing parameters to modify crystallinity and compare their antibacterial performance with that of commercially available CuO NPs. The antibacterial performance of CuO NPs-incorporated materials revealed significant differences in the efficacy between Gram-positive *S. aureus* and Gram-negative *E. coli*. The inclusion of CuO NPs led to a substantial decrease in bacterial live cells count, particularly for *S. aureus*, where reduction of up to 6 logs was obtained (Fig. 10a). Gram-positive bacteria, characterized with a thick peptidoglycan layer in their membrane, appear more susceptible to metal oxide NPs compared to Gram-negative bacteria, which possess an additional outer membrane that serves as a protective barrier.^51,52^ This observation is consistent with previous studies suggesting that this structural difference most probably contributes to the increased vulnerability of Gram-positive bacteria to metal oxide NPs.^51,52^ Additionally, the additive present in the polymers blend, which is more effective against Gram-positive bacteria,^50^ could further synergistically potentiate the initial antibacterial performance of the samples towards *S. aureus*.

Despite the strong bactericidal effect against *S. aureus*, the activity diminished significantly after three weeks of incubation in the simulated marine environment. This reduction could be attributed to the release and eventual depletion of CuO NPs over the prolonged exposure period.^53^ Although the different extrusion speeds during processing influenced the total release of copper ions (Table 3), materials prepared at lower speeds liberated less copper, possibly due to higher crystallinity,^54^ compared to those prepared at higher speeds with lower crystallinity. The 3 week incubation period in simulated marine conditions led to a complete loss of efficacy for all samples against *S. aureus*. This observation suggests that loss of activity was not solely due to the release of active agents but was also likely influenced by alterations in surface available CuO NPs under alkaline conditions, which could promote their dissolution.^55^ Conversely, the antibacterial performance against *E. coli* remained for all samples, regardless of the processing conditions. Interestingly, the initial killing effect on *E. coli* was lower for samples prepared at 10 kg h^−1^, which could be attributed to uneven NPs distribution within the polymeric matrix, resulting from the shorter residence time, or restricted contact with bacteria. These findings suggest that materials prepared under these conditions possess long-lasting antibacterial activity against Gram-negative pathogens such as *E. coli*. Optimizing processing parameters, including extrusion speed, is therefore critical to balance initial and long-term antibacterial performance across different bacterial types.

### Aquatic acute toxicity

**Fig. 11:**
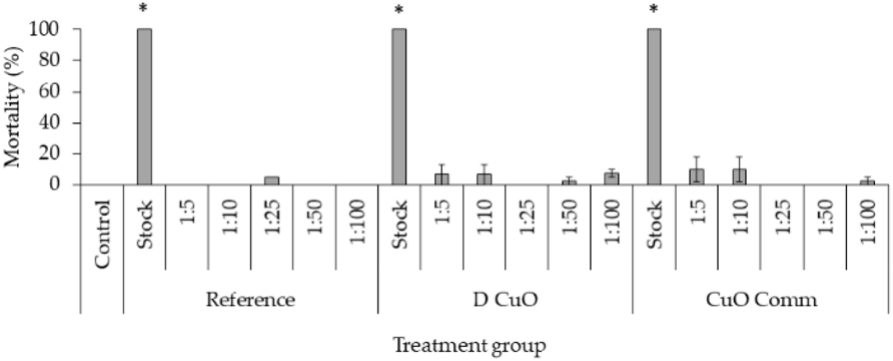
Mortality rates in 96-hpf embryos after exposure to three weeks leachates of reference, sonochemical CuO NPs (*i.e.*, “D CuO”) and commercial CuO NPs (*i.e.*, “CuO Comm”) mini-injected pieces. Values are given as mean ± SE, *n* = 3. * *p* < 0.05 *vs.* control (one-way ANOVA, *post hoc* Bonferroni).

The embryotoxicity of mini-injected pieces leachates investigated through the FET test demonstrated high embryotoxicity of undiluted mini-injected pieces leachates (“stock”), while already at the lowest dilution tested (1 : 5) no significant effect was remarked (Fig. 10). Regarding hatching, even though at the lowest dilutions (1 : 5 and 1 : 10) a slight delay was recorded at 72 hpf, all embryos hatched at the end of the exposure period (data not shown) (Fig. 11).

**Fig. 12:**
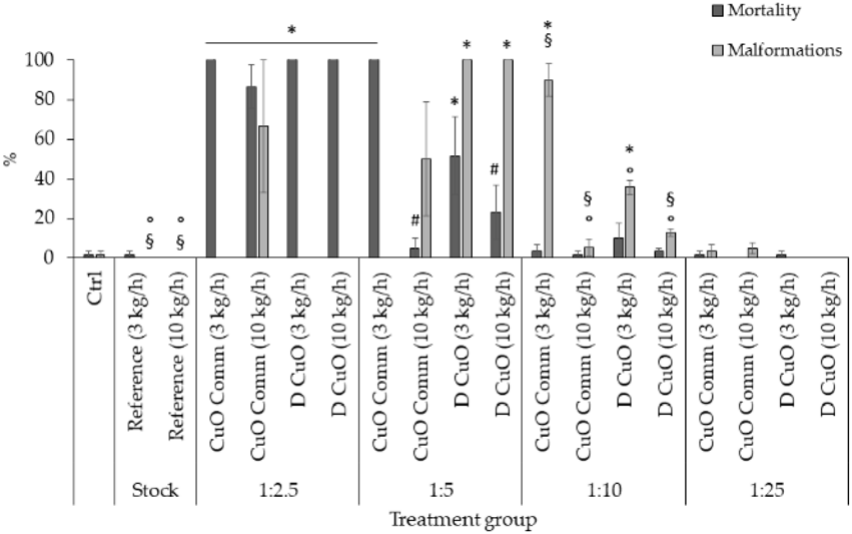
Mortality and malformation rates in 96-hpf embryos after exposure to four dilutions (1 : 2.5, 1 : 5, 1 : 10, 1 : 25) of three weeks leachates of reference, sonochemical CuO NPs (*i.e.*, “D CuO”) and commercial CuO NPs (*i.e.*, “CuO Comm”) monofilaments (3 and 10 kg h^−1^). Values are given as mean ± SE, *n* = 3. *p < 0.005 *vs.* control and reference, *p* < 0.05 *vs.* CuO commercial (3 kg h^−1^, 1 : 5), ^§^*p* < 0.05 *vs.* D CuO (3 kg h^−1^, 1 : 10), °*p* < 0.05 *vs.* CuO commercial (3 kg h^−1^, 1 : 10) (one-way ANOVA, post hoc Bonferroni).

FET test results of monofilaments' leachates evidenced a trend in mortality and malformation rates decreasing with increasing leachates dilutions (Fig. 12). Total lethality was observed among the embryos treated with undiluted leachates (data not shown), as well as in all 1 : 2.5 treatment groups, except for commercial CuO 10 kg h^−1^ (87%), where however the average malformation rate reached about 67%.

At 1 : 5 dilution a total lethality was found in embryos treated with commercial CuO 3 kg h^−1^, which was statistically significative compared to the control, to both reference treatments, commercial CuO 10 kg h^−1^ and D CuO 10 kg h^−1^. Additionally, the lethality among monofilaments extruded at 3 kg h^−1^ did not differ in a significative way at 1 : 5 dilution. Malformation rates were 100% in the two D CuO treatment groups and significantly different from control, reference and commercial CuO NPs groups.

Mortality and malformations dropped in dilutions 1 : 10 and 1 : 25, even if at 1 : 10 the malformation rate was significant for both the monofilaments co-extruded with CuO NPs at a pulling speed of 3 kg h^−1^, compared to all the treatments (Fig. 12).

To summarize, this study highlights a clear dose-dependent embryotoxicity of leachates from CuO NPs-enabled materials. In particular, the FET endpoints (*i.e.* mortality and malformation rates) decrease when diluting the leachates, ranging from a total mortality at the lowest dilution to a null effect on both lethality and malformations at the highest one (1 : 25). However, the levels of copper detected in the leachates (Table 3) are not sufficient to explain the observed toxicity, as they are below the concentrations found to be hazardous towards zebrafish early developmental stages.^56,57^ On the other hand, the pristine polymer does not induce toxicity *per se*.

Dilution 1 : 5 revealed the main differences among the effects induced by the treatments. Monofilaments extruded with commercial NPs at higher speed exhibited higher lethality, while monofilaments enabled with D CuO NPs induced greater malformations. Thus, the production processes affect leachate properties and bioavailability of toxic by-products. However, there does not seem to be a correlation between the registered toxicity outcomes and the amount of copper ions detected in the leachates (Table 3). Since higher toxicity would typically be expected with increased levels of copper ions,^58^ the adverse outcomes observed in this study appear unrelated to nano-enabling itself. Instead, they may derive from other components not addressed in this analysis. Indeed, other chemical species might be mobilized by the CuO doping and extrusion conditions of the PA monofilaments. In fact, these results lead to presume that by combining the polymeric matrix with the NPs, the nano-enabling may introduce changes in the leachate composition in terms of polymer, micro- and nanoplastics and/or additives possibly released, which may affect toxicity outcomes. For example, plasticizers have been reported to significantly induce mortality and severe malformations in developing zebrafish.^59^

Commercial CuO NPs have already been reported to impact the survival and to induce abnormal phenotypes in zebrafish embryos.^60^ Thus, our study highlights that toxicity seems to be associated to both type of and the extrusion speed. This leads to the conclusion that the safety of monofilaments nano-enabled with CuO NPs in the production of antibacterial/antibiofouling fish cage nets needs to be researched further using carefully selected manufacturing process parameters. Future research should focus on a comprehensive characterization of the leachates' physicochemical composition to fully elucidate these findings, in particular the possible changes in leachate composition and by-products (nanoparticles included) physical–chemical characteristics.

Given the high lethality and malformation rates observed at the highest dilutions, hatching rates were recorded from 48 to 96 hpf for the embryos exposed to the two lowest dilutions (*i.e.*, 1 : 10 and 1 : 25). Results are presented in Fig. 12.

**Fig. 13:**
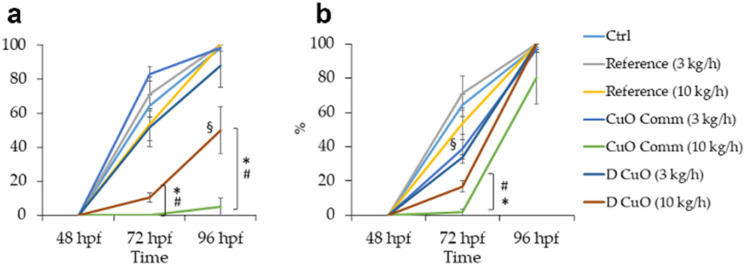
Hatching rates in 48 to 96-hpf embryos during exposure to dilutions 1 : 10 (a) and 1 : 25 (b) of three weeks leachates of reference, sonochemical CuO NPs (*i.e.*, “D CuO”) and commercial CuO NPs (*i.e.*, “CuO Comm”) monofilaments (3 and 10 kg h^−1^). Values are given as mean ± SE, *n* = 3. *p < 0.05 *vs.* control and reference, #*p* < 0.05 *vs.* D (3 kg h^−1^), ^§^*p* < 0.05 *vs.* CuO commercial (10 kg h^−1^) (one-way ANOVA, post hoc Bonferroni).

The heaviest effect on hatching was registered at the leachate dilutions corresponding to CuO commercial and D CuO NPs materials co-extruded at 10 kg h^−1^ extrusion speed, both at 72 and 96 hpf in the case of 1 : 10 (Fig. 13a) and particularly by the commercial NPs-extrusion monofilaments, while at 1 : 25 (Fig. 13b) the hatching delay was completely recovered at 96 hpf. Interestingly, the effect on the hatching endpoint is more evident for the leachates that have been resulted in higher amounts of copper ions (Table 3). This is consistent with the literature data, in which the copper ability to interfere with zebrafish embryos hatching is reported.^61,62^ The effect of hatching delay induced by metal oxide NPs has already been observed in zebrafish early stages of development and among the underlying mechanisms that have been proposed there is the hatching enzyme (ZHE1) inhibition by the dissolved metal ions.^63^

## Conclusion

This work demonstrates the successful incorporation of CuO NPs into the thermoplastic matrix of PA6 fish cage nets to endow them with antimicrobial and anti-biofouling properties. Through the unique antimicrobial properties of CuO NPs, the manufactured nets showed significant bactericidal activity against *S. aureus* and *E. coli* and maintained their effectiveness even after prolonged exposure to simulated marine conditions. Furthermore, fine-tuning the crystallinity of the PA6 material during production reduces copper ion leaching, which can be a significant contributor to aquatic ecotoxicity. These findings highlight the potential of CuO-enabled PA6 monofilaments as convenient solutions for mitigating biofouling challenges in aquaculture settings. However, the embryotoxicity investigated, which depends on dose and material manufacturing parameters, highlights the need for careful manufacturing protocols to minimize leachate toxicity. Nanoengineering of fish cage nets coupled with proper nano-ecotoxicity assessments could be contributesignificantly towards safer aquaculture with minimal impact on aquatic ecosystems.

## Ethical statement

All experiments were performed on embryos within 120 hours post fertilization (hpf), therefore they were not subject to the rules on animal testing according to European and Italian directives (European Commission, Directive 2010/63/EU of the European Parliament and of the Council of 22 September 2010 on the protection of animals used for scientific purposes, Official Journal of the European Union, 2010, L276).

## Author contributions

Conceptualization, Mika Paajanen, Tzanko Tzanov, Ilana Perelshtein; methodology, Milad Mosallaei, Pekka Laurikainen, Chandrahaasan K Soundararajan, Kristina Ivanova, Eva Ramon, Beatrice Negrini, Christian D'Abramo, Patrizia Bonfanti, Anita Colombo, Paride Mantecca; investigation, Milad Mosallaei, Pekka Laurikainen, Ella Kärkkäinen, Kristina Ivanova, Eva Ramon; resources, Tzanko Tzanov, Paride Mantecca; data curation, Milad Mosallaei, Kristina Ivanova; writing—original draft preparation, Milad Mosallaei, Ella Kärkkäinen, Kristina Ivanova, Beatrice Negrini; writing—review and editing, Milad Mosallaei, Ella Kärkkäinen, Pekka Laurikainen, Eetta Saarimäki, Mika Paajanen, Ilana Perelshtein, Kristina Ivanova, Eva Ramon, Tzanko Tzanov, Beatrice Negrini, Patrizia Bonfanti, Anita Colombo, Paride Mantecca.

## Conflicts of interest

There are no conflicts to declare.
